# Modeling obesity in complex food systems: Systematic review

**DOI:** 10.3389/fendo.2022.1027147

**Published:** 2022-10-13

**Authors:** Anita Bhatia, Sergiy Smetana, Volker Heinz, Joachim Hertzberg

**Affiliations:** ^1^ Food Data Group, German Institute of Food Technologies (DIL e.V.), Quakenbrück, Germany; ^2^ Knowledge-Based Systems Research Group, Institute of Computer Science, University of Osnabrück, Osnabrück, Germany; ^3^ Plan-Based Robot Control German Research Center for Artificial Intelligence, Osnabrück, Germany

**Keywords:** obesity, complex system, simulation model, machine learning, statistical methods, computational models, system dynamics

## Abstract

Obesity-related data derived from multiple complex systems spanning media, social, economic, food activity, health records, and infrastructure (sensors, smartphones, etc.) can assist us in understanding the relationship between obesity drivers for more efficient prevention and treatment. Reviewed literature shows a growing adaptation of the machine-learning model in recent years dealing with mechanisms and interventions in social influence, nutritional diet, eating behavior, physical activity, built environment, obesity prevalence prediction, distribution, and healthcare cost-related outcomes of obesity. Most models are designed to reflect through time and space at the individual level in a population, which indicates the need for a macro-level generalized population model. The model should consider all interconnected multi-system drivers to address obesity prevalence and intervention. This paper reviews existing computational models and datasets used to compute obesity outcomes to design a conceptual framework for establishing a macro-level generalized obesity model.

## 1 Introduction

Over the past two decades, there has been a significant rise in the prevalence of obesity, which has gradually turned into a global epidemic. Obesity is a global public health and economic issue that harms people’s physical and mental health, reduces their quality of life and life expectancy, and significantly increases the cost of healthcare systems (1). There are multiple causes and effects that contribute to obesity. Obesity is a multidimensional, systemic issue that affects a variety of domains, including social interactions, infrastructure, environment, and biology (2). Due to obesity’s global scope, heterogeneous drivers interacting in non-linear ways, and the lack of a single solution for variation in outcomes, a complex system is needed (3). A complex system model that can explain inter-connections and correlations of drivers and can examine non-linear dynamics, time-delay effects, multiple interactions, and feedback is required. Multiple authors used different system dynamic (SD) techniques and methods to model, predict, classify, and explain the prevalence of obesity and driver’s interconnection. Most models are designed with a specific question and focus on the limited number of links specific to a region or county. A holistic model explaining the indirect drivers of obesity in complex food system is missing. The multi-level sub-systems interconnections of obesity drivers must be investigated to derive general model, potentially able to clarify complex and indirect interconnections; Modeling multi-level model can become confusing and complex that results are no longer transparent, making validation impossible. To model a comprehensive general model, the multi-level sub-systems links of obesity should be explored, which can become so unwieldy and complex that results are no longer transparent, and validation becomes nearly impossible. So, currently, there is a need for a global level model, able to addresses the driver of interconnections and multi-level sub-systems of the obesity system (2). Vandenbroeck et al. (2) developed the qualitative obesity system map to understand the complex systemic structure of obesity using the causal loop model. The obesity system map can be used as a reference framework to design a conceptual, quantitative general obesity model. The obesity system map includes a variety of drivers, some of which have quantified causal relationships represented as mechanistic equations in the literature and others for which there is no quantitative data, such as environmental and social drivers. By using machine learning models (4), one can approximate the missing causative relationship and fill the gap to build a general complex obesity system model. The obesity system model has implications for future research aimed at early detection of obesity by hospitals and health professionals as well as to assist policymakers in testing interventions to analyze hotspots and where to intervene.

This review a) provides an overview of computational obesity models present in the scientific literature, including machine learning, agent-based, system dynamics, and simulation models that can explain the interconnections and non-linear dynamics between obesity drivers and b) analyzes the studies which have datasets and were reproducible c) and determines different modeling techniques strengths and limitations. Such an analysis should determine the potential of different models in defining and tracing the non-linear effects of key drivers of obesity. The review is aimed at the development of a conceptual framework for a global-level model by using and combining strengths of different modeling techniques.

The study begins with a bibliographic search and selection of literature for review, explaining the search terms and literature selection criteria for reviewing different models. We then examine the various modeling techniques of complex obesity systems and analyze their purpose, outcome, and limitations using the selected studies. The selected models are reviewed and discussed further, explaining the global level of a proposed framework and future development and application potential.

## 2 Methods

A systematic literature review (SLR) is an accessible and well-organized technique to define relevant research questions, keywords, and search phrases and study (5). An SLR analyses the research question and provides different methods for analyzing the problem thoroughly and broadly. The summary of the research methodology and SLR procedure is shown in following sub-section.

### 2.1 Overview

In order to conduct the SLR research and disclose the findings, this study adheres to the PRISMA guidelines (6). The SLR includes following tasks: define the search strategy and keywords and describe the inclusion and exclusion criteria. The review consists of computational models of obesity (6) which were written in English and published between January 1, 2002 and January 1, 2022 and had obesity-related search phrases and computational models.

After removing duplicate results, the potential studies were identified using relevant terms and keywords in electronic databases. Prior to text screening, the titles and abstracts of the records were first checked for inclusion and exclusion criteria for inclusion in the review.

### 2.2 Electronic searches

We searched scientific databases PubMed/Medicine (US National Library of Medicine) and Google Scholar (Google) for potential articles. We used a “backward and forward” search to determine the study references. To “go on” and find the articles cited in particular reviews, the google search engine was employed. The chosen studies serve as a starting point for finding articles that were relevant for our investigation. The logical search strategy based on Medical Subheading terms from PubMed: MeSH terms “obesity”, “overweight”, “obese”, “model”, “simulation model”, “system dynamics”, “agent-based model”, “ABM”, “machine learning” using query [(obesity[MeSH Terms] OR obese[MeSH Terms] or overweight[MeSH Terms]) AND (model[MeSH Terms] OR simulation model[MeSH Terms] OR system dynamic [MeSH Terms] OR agent-based model[MeSH Terms] or ABM[MeSH Terms] OR machine learning [MeSH Terms])]. The following were the chosen keywords for databases and Google scholar engine:

•(“Obesity” OR “Overweight” OR “Obese” OR “Adiposity”) AND (“Simulation model” OR “Simulation”)
•(“Obesity” OR “Overweight” OR “Obese” OR “Adiposity”) AND (“Agent based model” OR “ABM” OR “Agent-based model”)
•(“Obesity” OR “Overweight” OR “Obese” OR “Adiposity”) AND (“Machine learning” OR “Prediction”)
•(“Obesity” OR “Overweight” OR “Obese” OR “Adiposity”) AND (“System dynamics” OR “Model” OR “Computational model”) AND (“model”)

**Figure 1:**
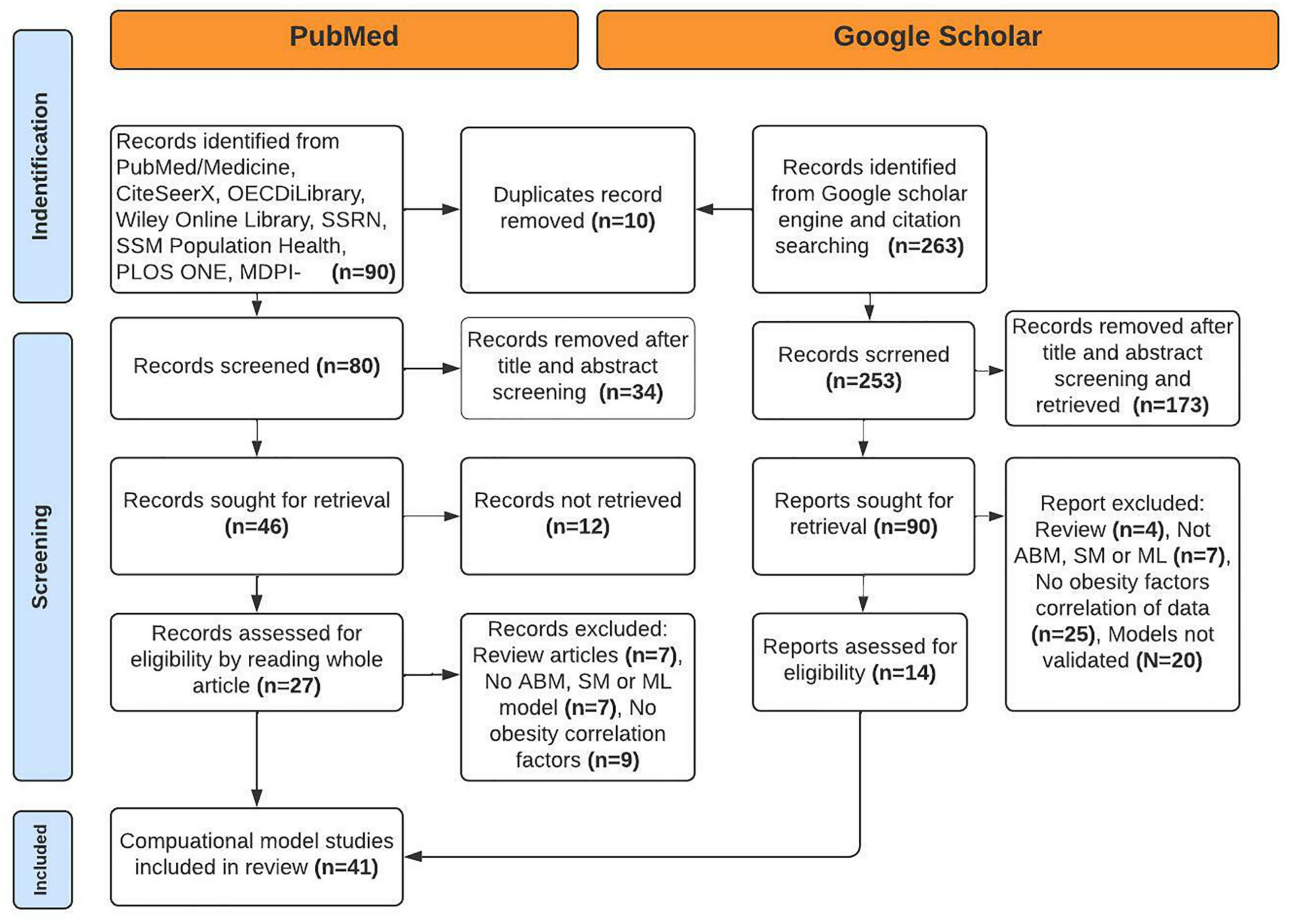
PRISMA diagram of identification and selection of studies for the review.

The search strategy included the databases, limiting research to 20 years (from May 2012 to May 2022). The studies were selected using a systematic and logical search strategy based on Medical Subheading terms from PubMed, IEEE, and query for Google Scholar engine as explained in **Figure 1**. After eliminating ten duplicates, the total articles examined on 6, Jan 2022 for the year (2002–2022) generated 136 hits.

### 2.3 Inclusion and exclusion criteria

To assure the relevance of the chosen articles to the study purpose of the research, inclusion comprises the characteristics that qualify the studies for inclusion, and exclusion includes the characteristics that disqualify the studies for inclusion. First, the article’s abstract and title was carefully reviewed to evaluate its suitability for the current SLR. After that, each study was examined to determine if it met the exclusion or inclusion criteria.

For this review, only articles about obesity and obesity computational models published in English from January 2002 and January 2022 were taken into consideration. The explicit inclusion and exclusion criteria used in the study summarized in **Table 1**.

**Table 1:** Inclusion and exclusion criteria.

Criteria	Principle
Inclusion	Studies published between January 2002- January 2022
	Studies written in English
	Studies that are complete and models have been validated on datasets
	Studies related to computational and machine learning models of obesity
	Studies explaining the relation of obesity drivers, prediction, trends, and prevalence of obesity
Exclusion	Duplicate studies
	Studies conducted in languages other than English
	Studies in which models were not validated against datasets
	Studies that did not explain the relation between obesity drivers using model
	Studies related to infants and genetic concepts
	Studies based on simulation model software

### 2.4 Study selection process

The primary goal of the selection procedure is to find relevant articles. Using the keywords provided above, an electronic search resulted in 353 articles. After deleting 10 duplicate articles with Mendeley’s reference management tool (Elsevier, UK), the results were narrowed down to 343 articles. We eliminated 185 studies from consideration by carefully reading abstracts, title and conclusions of studies and applying inclusion and exclusion criteria.

Exclusion criteria were used to assess the relevance of the remaining 136 articles to the research objectives. The manual and electronic search yielded 41 different computational models. Genetic factors and infant studies were not considered.

## 3 Modeling techniques for obesity

For simulating the complexity of obesity, there are a variety of methodologies available. Because of the depth and scale of the obesity epidemic, the model should be able to capture multi-level analysis when modeling the obesity complex system (7–9). Modeling at a single level does not allow to identify links and feedback loops between different levels (individual, population, national, and global levels) and its degree of influence on obesity outcomes. Second, the model should be able to capture individual heterogeneity as well as variation in adaptation over time. Finally, the model explains the problem and its mechanism to slow or reverse the epidemic (10). Given these requirements, computational and simulation models taken from the literature on obesity research and complexity science offers a set of useful tools.

### 3.1 System dynamics

System dynamics (SD) (11, 12) is a modeling technique in which a system is modeled using key drivers, flows and feedback loops to investigate the dynamics of a system under a set of scenarios, assumptions and datasets. The simulated computer model helps us determine under what conditions and how a part of the system might fail and identify the gaps in current knowledge. A simulation model has the following properties: a) stochastic and deterministic behavior, b) static and dynamic time series, and c) discrete and continuous data. Many simulation models based on food, physical activity, social environment, and economic trends have been implemented in the literature, as shown in **Table S1**.

#### 3.1.1 Mechanistic models of obesity

Hodgkin and Huxley (13) proposed the mechanistic modeling paradigm. It typically involves generating simplified mathematical equations of the causal mechanism by establishing a causal relationship between inputs and outputs based on observations of the phenomenon of interest (14). The mechanistic model can be implemented at both individual and population levels. Frerichs implemented the micro-level mechanistic model to assess the sensitivity of childhood obesity and social transmission rates. Fallah Fini et al. (15, 16) explored micro-level dynamics of Body Mass Index (BMI) distribution and prevalence to quantify the energy gap responsible for obesity in gender and racial population using mechanistic models and statistics.

#### 3.1.2 Markov simulation models of obesity

Markov models are stochastic models that can capture the distribution of attributes and model their dynamics (17, 18). Markov simulation model can recognize patterns, make predictions and learn the statistics of sequential data. Basu (19) forecasted BMI using Medical Expenditure Panel Survey (MEPS) data and a micro-simulated probabilistic population-level model. The model was validated using the National Health and Nutrition Examination Survey (NHANES) to project that obesity growth will continue in children aged 6-9, while overall obesity prevalence will remain constant. Individual-level Markov models explain how BMI trends differ across cohorts by taking demographics (age, race, height, BMI), socioeconomic and dietary composition, and individuals at household parameters. Ball et al. (20) created an individual-level dynamic discrete-time Markov micro-simulation model for estimating lifetime healthcare costs for healthy, obese, and smoker cohorts. The economic burden and productivity loss brought on by obesity were predicted by Lightwood et al. (21) using Discrete-time Markov cohort macro model.

#### 3.1.3 Statistical simulation models of obesity

Statistical modeling uses mathematical models and assumptions to study complex systems with arbitrary event flows at the inputs and distributions of time intervals of events. Sassi et al. (22) used the Log-Linear model to show BMI trends and the prevalence of obesity and overweight to access associated social gradients. In order to investigate the dynamics of obesity by body mass index (BMI), nutritional stage dynamics and trends in obesity by gender and socioeconomic status at the national, regional and sector level, Meisel et al. (23) developed a statistical model. Vreeman et al. (24) implemented a mathematical simulation model to understand better how food advertising and television contribute to obesity and determine the conditions under which changes in price and income affect body weight. Schroeter et al. (25) developed a micro-simulation economic model to explore eating behavior, food environment, and obesity interventions. The scalable mobility model based on the maximum utility and mobility framework can simulate individual-level weight change to show population-level weight change (26). Chen et al. (27) used a population-level model to see how population weight status and socioeconomic dispersion in longitudinal data projected the potential impact of socioeconomic interventions on obesity prevalence.

#### 3.1.4 Dynamic microsimulation models of obesity

Dynamic microsimulation models have a bottom-up approach and individual-level focus property. The microsimulation model assumes no assumptions about agents’ interaction. Using metabolic micro-simulated model one can achieve health goal by adjusting energy intake and physical activity (28). Using data from NHANES and British and Foreign School Society (BFSS) surveys, the network-based simulation model simulates how behavioural and social influences contribute to the spread of obesity and forecasts the efficacy of weight-loss interventions (29). To estimate how long will it take to reach the government’s goal of reducing the prevalence of obesity, Abidin et al. (30) simulated changes in children’s eating behavior using sub-models of food and energy intake, energy expenditure, and body composition.

### 3.2 Agent-based models of obesity

The complex dynamics are modeled in the agent-based model (ABM) by replicating the agent’s interactions and actions in the system’s environment in software code. The agents are positioned in a spatial environment with predetermined rules and initial conditions. At the individual systemic levels, interactions and decisions determine the outcomes. Complex systems can benefit from the computer simulation since it shows macro-level trends and patterns utilizing individual level outcomes and a bottom-up methodology (31). Different agent-based model studies are present in the literature on obesity. The studies were categorized according to characteristics, use cases, and variables involved, as shown in **Table S2**, with individual advantages and limitations. An ABM approach would enable the simulation of various systems at various scales while taking unique diversity into account (32).

#### 3.2.1 Mechanistic models

Different agent-based mechanistic models are present in the literature to understand the food decision-making process, food environment relation, and activity environment’s effects on obesity. Food costs, diet composition and food budget all factors that influence food decisions. Food inequalities can persist at the national level as a result of residential segregation, social networks, group preferences, and complex networks of social influence (33, 34). Food prices and store locations can influence low-income households’ diet quality, and food store spatial segregation promotes disparities in diet quality across income levels (35). The agent-based theoretical framework presented by Burke et al. (36) investigates the effects of decreasing food prices on weight gain, human metabolism, and social interaction. Rational addiction and variation in the self-control framework quantitatively predict the disproportionate growth in weight distribution using the discrete-time mechanistic model. Agents in the model a) compare their weight to the group’s averagely desired weight b) interactively and incrementally change their diets until they are less expensive than agent’s food budget (37). The relationship between the food reward environment and eating behavior and obesity can be explained by food reward hypothesis (38). To better comprehend how families, make restaurant selections based on socioeconomic, demographic, environmental, and nutritional characteristics, Li et al. (39) applied agent-based Huff model. to understand better how families, choose restaurants based on. Body weight can be affected by physical activity, location accessibility, leisure-time physical activity (LTPA), and obesity (40). The impact of social networks on adolescent body size, BMI, screen time, and sports participation are not well demonstrated (41).

Agent-based policy intervention model offers distinct insights into the dynamics of different obesity combat policy interventions. Zhang et al. (42) investigated the effects of societal norms, food price policies, and regulations influence on people’s eating behavior. Sugar-sweetened beverage warning labels and taxes can lower sugar-sweetened beverage consumption and body mass among youth (33, 43). Environmental and nutritional characteristics and proximity to food outlets (44) affect dietary habits. The food environment influences aggregated consumption habits and, food outlets open or closed, household income (45). The proximity of a walking destination encourages low-income neighbors to do physical activity, and the social context influences energy balance and obesity (46, 47). Increasing the community’s availability of neighborhood healthy food outlets, improving physical activity infrastructure, and higher school quality policies can help reduce body mass index disparities (48). Li et al. (49) looked at how mass media and nutrition education campaigns influence dietary habits and food intake. Household income, neighborhood income, school quality, food availability (neighborhood food environment), and exercise opportunity are the critical variables of complex obesity systemic structure. The key outcomes include educational attainment, socioeconomic status, social influence, physical activity, body mass index, cardiovascular health, and morbidity (50).

### 3.3 Machine learning models of obesity

By utilizing sensors, smartphone apps, electronic medical health records, and digital data, machine learning (ML) uses computer algorithms to automatically learn from experience and categorize risks and outcomes associated with obesity. As shown in **Table S3**, machine learning offers a novel approach to examining multivariate data and predicting the complex inter-relationships likely to cause obesity risks.

The machine learning algorithms provides a distinct picture of the current state of machine learning algorithms and data analysis. The learning algorithms can be used to characterize, adapt, learn, predict, and analyze data, thereby enhance our understanding of obesity and our ability for precise prediction. Nowadays, there is a massive amount of big data in the literature; gadgets, surveys, and data points alone have no value. Machine learning can decipher enormous amount of contradictory information and acquire new knowledge. Researchers used machine learning techniques like regression, Random Forest, Decision Tree, Convolution Neural Networks (CNN), and SVN to find connections between many causes of obesity. The inability of such models to explain the causal connection between the drivers is a major drawback for obesity modeling. To function with deep neural networks requires big data; the model will exaggerate biased outcomes due to its reliance on survey data.

Obesity levels can be detected using obesity-causing parameters, caloric intake, energy expenditure, physical activity, dietary and genetic disorders, socioeconomic factors, and anxiety or depression. Using computational intelligence methods, supervised (Decision Tree and SVN) for comparative analysis and classification, and unsupervised techniques like K-Means for clustering and validation of models (51). Using low-dimensional (Decision Tree, Random Forest, etc.) and high-dimensional ML approaches (SVN, CNN, etc.), statistical, and data visualization methodologies, we can identify potential risks associated with obesity models using different learning models. Regression analysis and data visualization approaches were used with publicly accessible health datasets from Kaggle, UCI, and Physio Net (52, 53) to comprehend how identified risk factors relate to weight change (54). Using food sales data, a population/country level model can be used to estimate the prevalence of obesity and identify the food categories that are most important for obesity prediction (55). Gender, age, and race/ethnicity were only marginally significant predictors of weight status, while physical activity was the most important factor (56). Different machine learning regression techniques can estimate the relative predictive relevance of BMI demographics and psychological, behavioral, and cognitive traits. Adolescent Brain Cognitive Development (ABCD) study (57) explained the role of fixed and potentially modifiable variables of the obesity system map and BMI datasets.

According to the study, social problems and screen time correlated significantly linked with BMI and modifiable therapy targets (58). Country-level demographics, socioeconomic, environmental, and healthcare factors explain the heterogeneity in country-level obesity prevalence better than conventional epidemiological techniques, which consider only small number of preselected variables. The basis of interpretation can be greatly explained by machine learning models (59). Conditional Random Forests were used to find diverse set of social, physical activity, and food characteristics that make up the obesogenic environment. Geographically co-occurring risk variables can be accessed using machine learning approach that is data-driven yet non-parametric (60). Using deep learning Convolution neural networks with Google Static Maps API images, one can analyze the link between features and obesity incidence based on built environment information. These algorithms extract relevant environment features, and regression can be used for quantifying the association (61).

## 4 Discussion

Computational models enable system-level thinking, modeling techniques, and tools to be used to study obesity as an integrated system and to explicitly model the complex system’s dynamics as well as non-linear and circular causality. Several reviews and studies available in the literature discuss the possible techniques for developing an obesity system model. According to Hammod et al. (62), complex systems and system modeling methodologies offer a promising field for researching complex dynamics of obesity. Levy et al. (63) summarize existing simulation models of obesity and the strengths and weaknesses of these models to suggest future research directions. Xue et al. (64) study the applications of system modeling in obesity. To address all essential aspects of obesity and highlight the most significant gaps and overlaps, Morshed et al. (65) summarize system dynamics and agent-based models. The machine learning, ABM, and SM models can predict childhood and adolescent obesity to assess obesity as a worldwide epidemic (2, 4, 65, 66). DeGregory et al. (66) review provides a unique overview of data analysis and machine learning methods explicitly applied to obesity. Vandenbroeck et al. (2) provide the casual loop system dynamic that explains all the possible drivers of obesity and their interconnections but is qualitative in nature with no real datasets for validation. In this review, we made an attempt to identify the majority of recent computational models that function on real-world data sets and provide quantified output to quantify the energy surplus that causes obesity.

Our review of the literature generated several key findings. First, during the past 20 years, obesity research mostly relied on SM and ABM. Most of the models implemented in SM and ABM were designed to answer specific question. Machine learning models can emerge as a valuable tool to deal with high-dimensional data (66–68), due to their high predictive power, ability to model complex, non-linear relationships between variables, and capacity. The application of computational models can cover multiple domains of obesity ranging from human metabolism to behavior, environment, activity, and social influence, allowing for a general view instead of focusing on individual pieces of the system. The SM based Markov simulation (16, 19–21, 62, 69, 70) statistical, network-based (29) and mechanistic (22, 23, 26) simulation models have been used in the literature to estimate the healthcare cost due to obesity outcomes; to predict the BMI distribution; and to stimulate metabolism, eating behavior, environment relationship, to quantify the energy balance responsible for obesity. An agent-based model (ABM) provides a platform to model interactions between agents to simulate social behavior, network structure, and interventions.

Second, computational models were implemented at the individual-level and population level. The individual-level models were used to understand the spatial-temporal dynamics of the epidemic. In contrast, the population level models were mechanistic models that relate individual-level responses to population density and structure, to study population dynamics. Seven of the 41 studies used empirical data to reproduce real-world scenarios at the country level, while the others were carried at the individual-level.

Third, most of the included studies used data from government sources such as National Health and Nutritional Examination Survey (NHANES 1971-2010) for studies based in the United States (US), the health survey for England (HSE), Longitudinal Cost and Medicare Current Beneficiary Survey (MCBS 1992-2001), Medical Expenditure Panel Survey (2001-2005) datasets, World Bank Data and country-specific health, and nutritional statistics.

This review demonstrated that analyzed methodologies have limits as shown in **Table 2**, and can be addressed by symbiotically combining their strengths and techniques into a viable solution for bridging the gaps. We can implement the quantified causal system that can explain the interconnection and causation between the drivers and can explain the indirect drivers (emergence properties) linked to the physical phenomenon of obesity.

**Table 2:** Advantages and limitations of simulation modeling techniques of a complex system.

Model	Advantages	Limitation
Markov simulation model	Markov models can capture attribute distributions and model their dynamics	Presume typically low-dimensional data space, which make them less versatile
	Straightforward to implement in standard software packages	Unable to deal with complex state transition such as path dependence and individual learning
Mechanistic model	Logic principles combined with deductive reasoning allow extrapolation to predict behavior that is not present in data	Data that span multi-space and time scales cannot be handled
	Establish a causal relationship between inputs and outputs	Can handle only small datasets
		No consideration of patterns and non-predictive in nature
		It is challenging to incorporate information from multiple spaces and times
Statistical model	Can understand any pattern in data	Applicable to quantitative data and results might be misleading
	Comprehensive statistical analysis is less subject to bias	Cannot apply to homogeneous data
	Results inform better decision making	Deals with groups and aggregates only
Microsimulation model	Using computer simulation, macro-level trends and patterns can be generated, making them suitable for complex system modeling	Microsimulation assumes no interaction between components
	Individual-level focus property allows substantial diversity and heterogeneity among agents	It is challenging to measure social influence and transmission
Agent-based model	Using computer simulation, the bottom-up approach can generate macro-level trends and patterns	Not beneficial when dealing with homogeneous data as ABM focuses on the individual difference and how this difference contributes to system patterns
	Allow substantial diversity and heterogeneity among agents	Computationally expensive
	Capable of incorporating spatial contexts and feedback dynamics	The model must be built at the proper degree of description and with the appropriate level of details to serve its purpose
	Can examine non-equilibrium dynamics and focus on mechanisms	The general-purpose model cannot work
Machine learning model	Inductive capability- from past data, one can identify patterns in the data	Require large datasets
	Can tackle multiple spaces and time scales	Can only predict based on patterns in the provided data

### 4.1 Limitations of the studies

System modeling techniques have certain limitations as shown in **Table 2**. Firstly, while building a detailed and comprehensive obesity system map framework, the model can become so sophisticated that the results will no longer be transparent, and validation will become almost impossible. To overcome this limitation, the whole complex system can be divided into sub-systems (2). Obesity interventions vary across populations and regions and are sensitive to assumptions and settings. As a result, SD has limitations in guiding global future intervention development.

Second, the models depended on survey data, prone to bias due to self-reported datasets. Only a few model results are reproducible and documented in selected studies (26, 35, 43, 46, 54, 55, 71), the remaining studies were either slightly or moderately reproducible. Available models were created with a single or a few questions in mind and occasionally could not incorporate demographic data directly. They cannot scale to other variables and sub-populations, as described in system dynamics limitations and machine learning techniques **Tables S1–S3**. The global system model simulated counterfactual comparison, and the lack of empirical data and uncertainty of assumptions remains a significant challenge.

### 4.2 Matching existing models to the theoretical framework of current study

One of the most significant challenges in developing and implementing the obesity model is the complexity of the systems and the scarcity of datasets for all the drivers). One feasible option for modeling obesity’s complex structure is to use a modular approach. When studying multi-level feedback and interactions, a modular approach allows for separate analysis of each system and level, allowing easy integration. Each sub-system (for example, physical activity patterns, food intake and production, social and individual psychology, environment, and physiology) would have its module, with mechanisms theory and impact paths incorporated (3). We can use both machine learning and the SM technique to investigate the system dynamics of obesity. SM allows for the integration of data from wide range of disciplines, including statistics, epidemiology, biology, nutrition, and so on. Data-driven machine learning approaches can investigate the driver’s relationship and trends (72). Mechanistic model equations (7) can interpolate food production and consumption drivers (purchasing power, nutritional value, alcohol intake, portion size) to energy balance. Some drivers in the model’s psychology sub-system, such as social tolerance of fatness and food literacy, are not quantitative, and data-driven machine learning models (73) can be used to approximate the relationship between the drivers.

**Figure 2:**
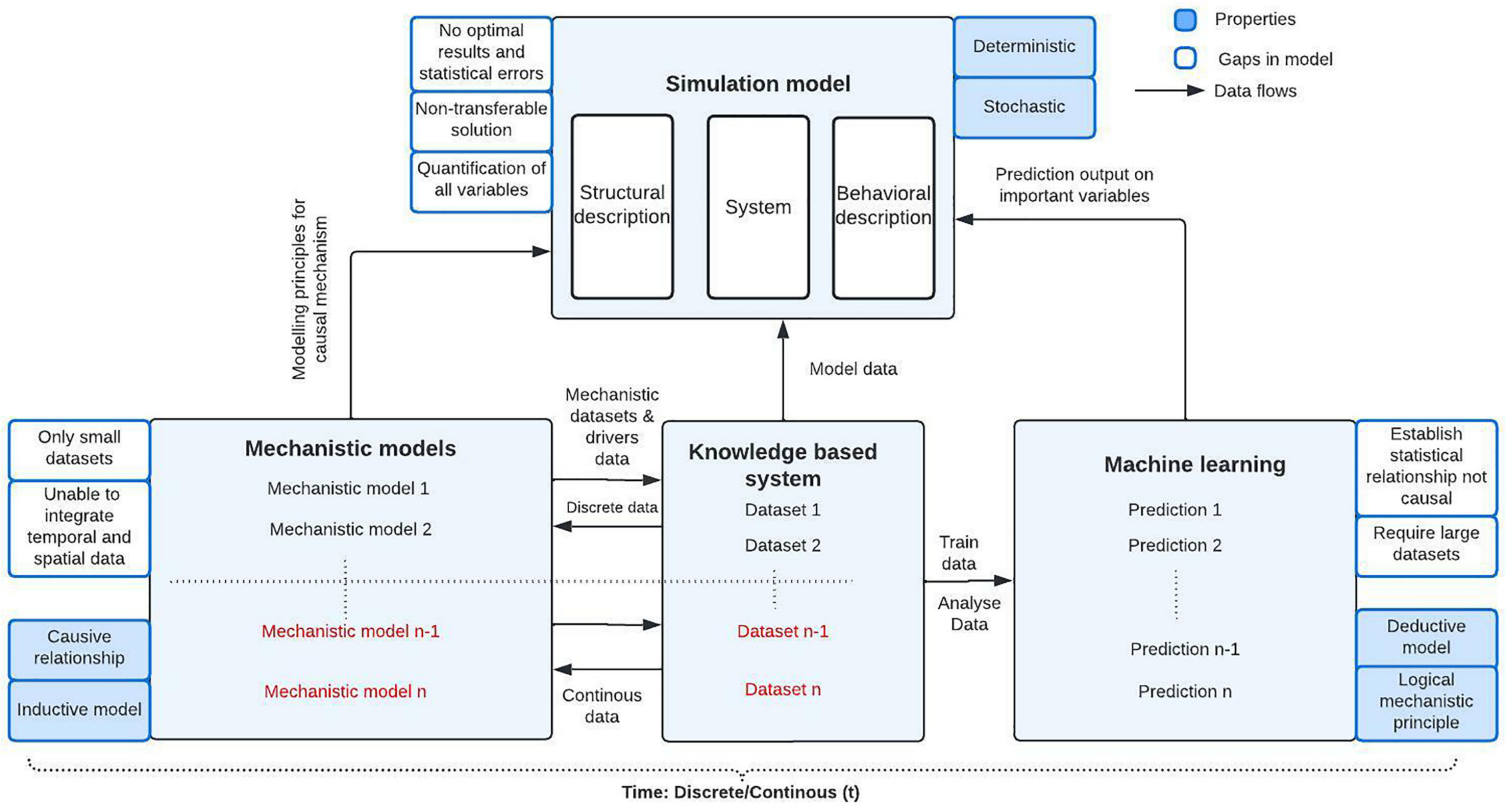
Framework of complex system model of obesity (national-global level of application).

These models are proposed to be combined to form a framework (**Figure 2**) to model the quantitative obesity systemic map (74). To implement the framework, the complex obesity system model needs to be divided into sub-systems of Food production and consumption, Psychology, Physical and Environment activity. Then, collected data from the available databases and literature would be used to define the level of importance of drivers related to obesity sub-systems along with the mechanistic mathematical equations that explain the causal relations between the drivers. Using the available datasets from NHANES and MPES etc. and mechanistic equations, new datasets can be generated and can be a part of a knowledge-based system.

The machine-learning models will be included in the framework as the certain driver’s causal relation connections are missing. Machine learning techniques can fill the gap. Mechanistic models supply the causality that machine learning methods lack, and machine learning models supply inductive capability and handle multi-space and time scale data to approximate the relationships between drivers using past observations.

The knowledge-based system consists of discrete and continuous data of obesity can be used to train and analyze the machine learning model. The ensemble mechanistic machine learning model allow to quantify the correlation between the drivers and predict the obesity emergence dynamics and the driver’s role in the prediction which can be possible using the application of Rule based explanations of machine learning and knowledge graphs (75). Machine learning can overcome the scalability limitation of mechanistic model. The predicted values will act as a behavioral description of the model, which can then be used to identify hotspots to intervene and predict future scenarios and assist policymakers in testing policies in the complex food system.

## Conclusions

Obesity is a multi-dimensional system problem. To design full capacity model to enhance obesity research and intervention, theoretical and practical issues with current computational models must be taken into consideration. Due to obesity’s non-linear dynamics and complex system effect, there is a need for emergence-focused design in complex system simulations to reproduce the multi-level emergence seen in the real world. The analysis of the literature indicated that both population-level data (weight status, socioeconomic position, physical activity, behavior and social network, and so on) and longitudinal data are required to assess the global level effect. Despite the limitations of individual simulation model, agent-based and machine learning models can fill missing gaps and function together symbiotically to model individual-level models. These individual-level models can be aggregated to global levels and estimate individual and population-level obesity dynamics. The complex hybrid system framework based on a synergetic combination of mechanistic, machine learning, and simulation model components may provide an innovative systematic approach to determine the complex interactions between obesity factors and fight the obesity epidemic.

## Author contributions

The study was conceptualized and designed by AB, SS, VH and JH. AB performed the literature review and wrote the first draft of the manuscript. JH and SS edited and proofread the manuscript. All authors contributed to the article and read and approved the submitted version.

## Funding

This research is partially funded by the German Federal Ministry of Education and Research (BMBF), in the frame of FACCE-SURPLUS/FACCE-JPI project UpWaste, grant number 031B0934A.

## Acknowledgments

The authors acknowledge the support of DIL e.V. (German Institute of Food Technologies) for the implementation of the study.

## Conflict of interest

The authors declare that the research was conducted in the absence of any commercial or financial relationships that could be construed as a potential conflict of interest.

## Publisher’s note

All claims expressed in this article are solely those of the authors and do not necessarily represent those of their affiliated organizations, or those of the publisher, the editors and the reviewers. Any product that may be evaluated in this article, or claim that may be made by its manufacturer, is not guaranteed or endorsed by the publisher.
