# Supplementary material for: Modeling obesity in complex food systems: Systematic review
Source: Front Endocrinol (Lausanne). 2022 Oct 13;13:1027147. doi: 10.3389/fendo.2022.1027147 (PMC9606209; doi:10.3389/fendo.2022.1027147)
Supplement: Supplementary file 1 [file Table_1.docx]

Supplementary Material

**Table S1: Simulations model of obesity**

| Reference | Objective | Model | Primary Outcome | Datasets | Limitation | Model Type |
| --- | --- | --- | --- | --- | --- | --- |
| Wang et al. (2007) | Forecast BMI distribution along demographics based on race, sex, and birth cohorts. | Discrete-time Markov model | BMI distribution | NHANES 1971-2004 | Change in mortality, age, and racial ethnicity were not considered, which causes misclassification and overestimation | Trends in obesity |
|  |  |  |  |  |  | Individual level model |
| Baal et al. (2008) | Estimate and compare the annual and lifetime medical costs associated with obesity and smoking cohorts | Discrete-time Markov simulation model | Disease healthcare cost  Medical costs for smokers | MCBS (1992-2001) | Causal relation between BMI and health care cost is unspecified. | Health outcome |
|  |  |  |  |  | Same cost and no transition for all risk factors | Individual level model |
| Schroeter et al. (2008) | To identify the conditions under which price and income change affect weight | Utility maximization framework | Energy accounting and food weight and exercise weight elasticity | NHANES (1963-1965, 1999-2002)  CDC (2004,2002)  American Time Use Survey (ATUS) | Limited alternatives were considered to study food and price elasticity | Economic model intervention for obesity prevalence reduction |
|  |  |  |  |  | Overestimation of results during sensitive analysis and model |  |
|  |  |  |  |  | Provide relative intervention option, not the quantifiable one |  |
| Bahr et al. (2009) | Simulate how obesity spreads across social networks and predict the effectiveness of weight interventions | Network-based microsimulation model | Behavioural model and social influence of obesity | BFSS  CDC | Traditional weight loss and dietary interventions will not work | Social interaction |
|  |  |  |  |  |  | Individual level model |
| Lightwood et al. (2009) | Predict future economic burden attributable to obesity | Discrete-time Markov cohort macro model | Healthcare cost and productivity loss from 2020-2050 | NHANES (1971-2000) | Model ignores real human welfare cost and underestimates long-term consequences | Health outcome |
|  |  |  |  |  |  | Individual level model |
| Sassi et al. (2009) | Forecast prevalence of obesity and access associated social gradients (social-economic condition, gender, age) | Log-Linear model | Trends in obesity | NHANES | Exploratory data based on assumptions | Trends in obesity |
|  |  |  |  |  | Key determinants of change are not known | Individual level model |
|  |  |  |  |  | Unavailability of diet, physical activity, and social-economic factor data in multimodal |  |
| Veerman et al. (2009) | To estimate how much would be limiting TV food advertisements reduce obesity | Mathematical simulation model | Obesity prevalence and average BMI | NHANES (2003-2004)  CDC (2000) | Uncertainty in the size of effects and lack of quantified data. | Individual level model |
|  |  |  |  |  | Overestimation of results due to data biases and linear relationship between food advertisement and obesity. |  |
| Basu, Anirban (2010) | Predict the distribution of BMI for children and adults according to age, sex, and race | Discreet-time Markov macro model | Forecast distribution from 2004-2014 | MEPS (2001–2005)  NHANES 2005–2006 | Bias due to self-reported data | Trend in obesity |
|  |  |  |  |  | Transitions variation for children every year limits prediction | Population-level model |
| Rahmandad et al. (2012) | Modeled individual level weight change to simulate population level weight change | Scalability  Mobility Model | Population-level trend of obesity and capture energy balance and weight change | NHANES (2000-2009) | Fit between data and model can be improved. | Eating behaviour and food environment |
|  |  |  |  |  | Limited time series data | Individual level model |
| Frerichs et al. (2013) | To assess the sensitivity of childhood overweight and obesity and social transmission rates | Mechanistic model and Two-way sensitivity analysis | Obesity prevalence | NHANES (2009-2010) | Non-predictive model due to deterministic nature. | Social influence on BMI and physical activity (PA) |
|  |  |  |  |  | No statistical significance between intervention combination and consideration of patterns and outcomes that points to potential gaps. | Individual level model |
| Abidin et al. (2014) | To simulate eating behavior, identify how long it will take to reach the government's targeted obesity prevalence. | Mechanistic | Medical expenditure, productivity loss and diet healthfulness | Health Survey of England | Lack of historical data on energy intake and physical data in the survey. | Obesity prevalence |
|  |  |  |  |  | Wrong assumption of similar eating behaviour for all ages and gender and limited number of variables tested in optimization stage | Eating behaviour |
| Basu et al. (2014) | Estimate change in calorie intake and physical activity to achieve Healthy People2020 objective | Stochastic, Discrete time, individual-level microsimulation model | Forecast BMI prevalence | NHANES (1999-2010)  NIH model | Used data of NHANES, was subjected to surveys, recall and acceptability biases | Population level model |
|  |  |  |  |  | Model did not capture all metabolic effect of physical activity. |  |
| Fallah-Fini (2014) | To quantify the energy imbalance gap in gender/racial population | Micro-level mechanistic models and statistics | Average body mass index and weight | NAHNES (1971-2010) | Unidentified behavioural and environmental factors that cause energy imbalance gap (EIG) | Body weight dynamics |
|  |  |  |  |  | Model applied on only one sub-population at a time | Population level model |
| Chen et al. (2018) | To study the country-level dynamics and influence between population weight and socio-economic status | Statistical model | Obesity prevalence | Medical expenditure Panel Survey (2001-2011) | Model relies on assumption- past dynamics and interactions would continue | Social influence and obesity-related outcome |
|  |  |  |  |  | Body weight and employment status were self-reported data | Population level model |
| Meisel et al. (2018) | Analyse the nutritional stage dynamics of the urban Columbian population. | Statistical model | Nutritional transitions in low and middle-income countries | DHS (2005,2010)  DANE (1985-2020)  World Bank Data (1960-2014)  WHO child standards and cut-off points | Estimations based on survey data cause biasness  Same mortality rate used for all BMI categories and transference rate between BMI categories by age and socio-economic status was not considered | Population based model  Nutritional stage by socio-economic status |

NHANES: National Health and Expenditure Survey

CDC: Centers for Disease Control and Prevention

MEPS: Medical Expenditure Panel Survey

WHO: World Health Organisation

MCBS: Medicare Current Beneficiary Survey

DANE: Columbian National Department of Statistics

DHS: Demographic Health Survey

MCBS: Medicare Current Beneficiary Survey

**Table S2: Agent-based model of obesity**

| Reference | Objective | ABM Class | Primary outcome | Datasets | Limitation | Model Information |
| --- | --- | --- | --- | --- | --- | --- |
| Burke et al. (2007) | To determine the relationship between food prices and obesity rates | Mechanistic | Body weight and obesity | NHANES (1976-2000) | Lack of knowledge of weight-metabolic curve. | **Dependencies**: Follow the average |
|  |  |  |  |  | No quantitative information about self-control and weight gain variation. | **Feedback loop**: Social influence |
| Hammond et al. (2007) | Analyse physiology of dieting and socially influenced weights | Mechanistic | BMI and weight distribution | Hypothetical data | Based on hypothetical data | **Dependencies**: Follow the average |
|  |  |  |  |  |  | **Feedback loop**: Social influence |
| Auchincloss et al. (2011) | To explore the role of income disparities in diet | Mechanistic and policy | Income disparities in diet | Household shopping behaviour and economics dataset from literature | Insufficient data for policy recommendations | **Feedback loops**: Store, store closure, food choice |
|  |  |  |  |  |  | **Spatial sensing**: 50*50 grid, rank - distance to store stratified by income |
| Brown et al. (2011) | Simulate the effect of community safety in adult daily walking behavior | Policy | Walking behaviour | National Household Travel Survey (2001)  Ancon government census data | Gender differences was not modelled, and aesthetic quality was assumed constant | **Spatial sensing**: City of 64km2 (800*800) grid space |
|  |  |  |  |  | Discrepancies between model data and model output | **Feedback**: Attitude toward walking, density of other walkers and total distance walked by person |
| Shoham et al. (2012) | To examine the hypothesis that social influence on adolescent body size is independent of friend selection | Mechanistic | Patterns of adolescent obesity | National Longitudinal Study of Adolescent Health | Self-reported data on BMI, screen time, and frequency of playing sports | **Feedback**: Peer influence |
|  |  |  |  |  | Model was deterministic and could not handle continuous outcomes | **Dependencies**: Social influence and screen time |
| Giabbanelli et al. (2012) | Explore the interaction of social networks and environmental factors and their influence on obesity | Policy | Eating behavior and physical activity | National Longitudinal Survey of Youth (1979) | Numerous data gap |  |
|  |  |  |  |  | Non-scalar model |  |
| Powell-Willey et al. (2012) | Quantify the impact of crime on physical activity and obesity | Mechanistic | Obesity prevalence and leisure-time physical activity | US Census Bureau  NHANES (2013-2014)  Washington Crime Surveillance -2014  Washington DC. Cardiovascular Health and Needs Assessment | No accountancy for the difference in LTPA according to location | **Spatial sensing**: Radius from home 0.5 miles if walking; 2.5 miles if driving. |
|  |  |  |  |  | Assume that compensatory eating did not occur. | **Dependencies**: Baseline exercise probability |
| Orr et al. (2014, 2016) | To investigate the effect of 3 policies to remove disparities in BMI by a) improving education quality, b) access to healthy food c) physical activity infrastructure. | Policy | Healthy diet (Healthy eating index) | National health interview Survey | No gender and geography information available for agents. | **Dependencies**: Social influence |
|  |  |  |  |  | Time frame of scenario was very huge | **Feedback loops**: School quality, education level, household income, residential morbidity |
|  |  |  |  |  |  | **Spatial sensing**: Distance |
| Zhang et al. (2014) | Examine policy on different unhealthy eating behaviour | Policy | Obesity and daily consumption of serving and fast food | Food Attitudes and Behaviours Survey 2007 | Lack of data regarding the extent to which friends influence people. | **Dependencies**: Follow the average |
|  |  |  |  |  | Lack of empirical information biases occur due to food advertisement | **Feedback loops**: Food choices, food environment |
|  |  |  |  |  | Assumption of constant population characteristics (age, gender, and education) | **Spatial sensing**: Buffer zone around home of 50-200 cells (representing 0.25 - 1.0 mile). |
| Blok et al. (2015) | To understand income inequalities in healthy food consumption | Policy | Income difference in fast food diets | Statistics Netherland  Eindhoven  GLOBE: Food behavioural data  Food outlets: Yellow Pages | Demonstrative model that doesn’t show a real-world scenario | **Feedback**: Food preferences |
|  |  |  |  |  |  | **Spatial** sensing: 10*10 m^2^ Depend on higher utility |
| Zhang et al. (2015) | Test the impact of social influence on overweight and obesity prevalence | Policy | Overweight and obesity prevalence | National Longitudinal study of Adolescent Health (2004-2005) | Ungeneralizable outside considered population | **Feedback**: Social norms (negative feedback) |
|  |  |  |  |  | Model could not expand beyond one simulated year |  |
|  |  |  |  |  | Predicting an individual agent's behaviour was not possible |  |
| Beheshti et al. (2016) | Examine the effect of food budget change on diet | Mechanistic | Proportion of calorific intake | NHANES  USDA Food and Nutrient Database for Dietary Studies  US Census Bureau and BLS  International Comparison Program (2005) | Single dominant price matrix | Diet change is incremental, so diet at t+1 largely depends on the diet at t. |
|  |  |  |  |  | Unable to assess decision making to get people who spent more than the average price required. |  |
|  |  |  |  |  | It cannot be applied to high-income population |  |
| Langellier, Brent A. (2016,2017) | To understand the complex relationship between residential segregation and social influence on diet preferences. | Mechanistic | Bubble gum chewing and sugar-sweetened beverages consumption model | NHANES (2007-2010)  American Community Survey (2010-2014)  Dun and Brad sheet (Food outlets-2012)  Retail food Environment Index, Wolstein, and Diamant (2011) | National data cannot present SSB consumption behaviour correctly. | **Feedback loop**: Social preferences |
|  |  |  |  |  |  | **Dependencies**: Social influence, Follow the average |
|  |  |  |  |  |  | **Spatial sensing**: Neighbourhood agents and agents in the same school |
| Li et al. (2016) | To investigate the effect of mass media and nutrition messages on food consumption | Policy | Dietary behaviour | Food Attitudes and Behaviour survey  New York Community Health Survey  US Census Bureau | Demographic data were not directly included | **Dependencies**: Taste preferences and health beliefs |
|  |  |  |  |  | All food outlets, stores, and social messages were not considered | **Feedback** **loops**: Social influence |
|  |  |  |  |  |  | **Spatial** **sensing**: Distance within 1 mile |
| Lee et al. (2018) | Examine the impact of health warning labels on obesity prevalence | Policy | Prevalence of obesity | NHANES (2010-2014) | Same efficacy for purchase labels irrespective of the type of store that could overestimate or underestimate the impact. | **Spatial sensing**: Geospatial grids for each city |
|  |  |  |  |  | Economic impact was not considered |  |
| Li et al. (2018) | To understand the relation between restaurant choice and socio-economic, demographic, environmental, and nutritional factors | Mechanistic | Restaurant choices | NEMS-Restaurant | Family income, parental education, and transportation mode data were not considered. | **Spatial sensing**: Healthy parameter  **Feedback** **loops:** Social influence |
|  |  |  |  |  | Age was not reflected in the model due to bias. | **Dependencies**: Follow the average |

NEMS: Nutrition Environment Measures Survey

GLOBE: Global leadership and Organizational Behavior Effectiveness

NHANES: National Health and Expenditure Survey

LTPA: Leisure Time Physical Activity

US: United States

USDA: US Department of Agriculture

BLS: Bureau of Labor Statistics

**Table S3: Machine learning model of obesity**

| **Reference** | **Objective** | **ML Models** | **Datasets** | **Limitation** |
| --- | --- | --- | --- | --- |
| Nau et al. (2015) | Use forest instead of tree ML algorithms to define obesogenic and obese-protective environments | **Statistical analysis**: Conditional Random forest  **Validation**: Out of Bag (OOB) | Geisinger Health System (2001-2012)  Info USA and Dun & Bradstreet^[[1]](#footnote-1)^  NAICS codes  ACS survey  Pennsylvania Department of Transportation | No clear indication of direction or intensity of association between a predictor and the supervisor |
|  |  |  |  | Unable to infer that people in the obesogenic environment were at higher risk |
|  |  |  |  | Limited scope of inferences to the relation between community constructs and average BMI |
| Maharana et al. (2018) | To examine the association of the built environment with obesity prevalence | **Extract features and quantify**: Convolution neural network Regression  **Validation and Testing**: Random samples | BRFSS survey  CDC data | Self-reported data cause biasness and over/underestimate the results |
|  |  |  |  | Differences in timing of obesity data and satellite data and lack of datasets |
| Scheinker et al. (2019) | To identify country-level factors associated with obesity | **Statistical analysis**: Regression: Single, multivariate, LASSO, Gradient Boosting Machine- Random forestBayesian  **Validation and testing**: 5-fold cross-validation | BRFSS survey data  American Community Survey  CDC Diabetes Interactive Atlas and mortality data  US Census and USDA ERS Food Environment Atlas | Based on self-reported datasets that could cause bias and overestimate the results. |
|  |  |  |  | Unable to establish a causal relationship between variables due to its black box nature. |
|  |  |  |  | Individual risks were not captured. |
| Cervantes et al. (2020) | To estimate obesity level using computational intelligence and data timing | **Comparative analysis and Classification:** Decision tree, SVM  **Validation**: K-Means clustering  **Testing and Training**- Cross-validation | WHO Obesity level and survey data | Biasness due to self-reported survey data |
|  |  |  |  | Computational model learns from data – any inaccuracies in data reflected in results |
| Chatterjee et al. (2020) | To know how to use different ML methods to identify potential risk factors of obesity/overweight | **Comparative analysis and Classification:** Regression, SVM Classifier, D-Tree, Naïve Bayes  **Training and testing**: K-fold cross-validation | Kaggle^[[2]](#footnote-2)^ , UCI^[[3]](#footnote-3)^ , Physio Net^[[4]](#footnote-4)^ repositories | Dataset was small and not from the same source |
|  |  |  |  | Individual level model and didn’t use real world data |
| Dustan et al. (2020) | To predict country-level obesity prevalence based on national sales of some food and beverages categories | **Exploratory analysis**: PCA, SVM  **Feature selection**: SVM, Random Forest, Extreme Gradient Boost  **Validation**: Leave-One-Out Cross-Validation, Random forest | Euromonitor dataset  Percentage of obese adults in 2008 | Performance of the algorithm may vary country by country according to selected food categories. |
|  |  |  |  | Unable to determine whether diet composition or sedentary behaviour was the cause of obesity prevalence. |
| Gray et al. (2020) | To estimate the relative correlation between BMI and demographic, psychological, behavioural, and cognitive variables of obesity | **Exploratory analysis**: LASSO regression  Elastic net regression  Linear regression | ABCD Study^[[5]](#footnote-5)^  NIMH data | Unable to determine causality due to cross-sectional data |
|  |  |  |  | Lack of energy intake and expenditure data and lack of granularity to access multiple aspects. |
| Cheng et al. (2021) | Examine the relationship between physical activity and weight status using machine learning and statistical methods | **Classification and comparison**: Naive Bayes, RBF, Local k-NN, CVR, D-Tree, multi-objective evolutionary fuzzy classifier, Random tree, Multilayer Perceptron, Logistic regression | NHANES (2003-2006) | Not all physical activities were considered. |
|  |  |  |  | Examined only weight status, not body composition |

LASSO: Least Absolute Shrinkage and Selection Operator

D-Tree: Decision tree

SVM: Support Vector Machine

ABCD: Adolescent Brain Cognitive Development

PCA: Principal Component Analysis

NIHM: National Institute of Mental Health Data Archive

BRFSS: Behavioral Risk Factor Surveillance System

CDC: Centers for Disease Control and Prevention

ACS: American Community Survey

US ERDS: US Department of Agriculture Food Environment Atlas

NPI: National Provider Identification

NHANES: National Health and Nutrition Examination Survey

RBF: Radial Basis Function

API: Application Programmable Interface

k-NN: k-Nearest Neighbors

CVR: Classification via regression

1. Dun & Bradstreet: <https://www.dnb.com/> [↑](#footnote-ref-1)
2. Kaggle: <https://www.kaggle.com/data> [↑](#footnote-ref-2)
3. UCI: <https://archive.ics.uci.edu/ml/index.php> [↑](#footnote-ref-3)
4. Physio Net: [https://physionet.org/about/database](https://physionet.org/about/database/)/ [↑](#footnote-ref-4)
5. ABCD study: <https://abcdstudy.org> [↑](#footnote-ref-5)
